# Acute Kidney Injury Patterns Following Transplantation of Steatotic Liver Allografts

**DOI:** 10.3390/jcm9040954

**Published:** 2020-03-30

**Authors:** Caroline Jadlowiec, Maxwell Smith, Matthew Neville, Shennen Mao, Dina Abdelwahab, Kunam Reddy, Adyr Moss, Bashar Aqel, Timucin Taner

**Affiliations:** 1 Division of Transplant Surgery, Mayo Clinic, Phoenix, AZ 85054, USA; 2 Division of Anatomic Pathology, Mayo Clinic, Phoenix, AZ 85054, USA; 3 Instructor in Biostatistics, Mayo Clinic College of Medicine, Phoenix, AZ 85054, USA; 4 Division of Transplant Surgery, Mayo Clinic, Jacksonville, FL 32224, USA; 5 Division of Nephrology, Mayo Clinic, Phoenix, AZ 85054, USA; 6 Division of Transplant Hepatology, Mayo Clinic, Phoenix, AZ 85054, USA; 7 Division of Transplant Surgery, William J von Liebig Transplant Center, Rochester, MN 55902, USA

**Keywords:** acute kidney injury, allograft steatosis, lipopeliosis

## Abstract

Background: Steatotic grafts are increasingly being used for liver transplant (LT); however, the impact of graft steatosis on renal function has not been well described. Methods: A total of 511 allografts from Mayo Clinic Arizona and Minnesota were assessed. We evaluated post-LT acute kidney injury (AKI) patterns, perioperative variables and one-year outcomes for patients receiving moderately steatotic allografts (>30% macrovesicular steatosis, *n* = 40) and compared them to non-steatotic graft recipients. Results: Post-LT AKI occurred in 52.5% of steatotic graft recipients versus 16.7% in non-steatotic recipients (*p* < 0.001). Ten percent of steatotic graft recipients required new dialysis post-LT (*p* = 0.003). At five years, there were no differences for AKI vs. no AKI patient survival (HR 0.95, 95% CI 0.08–10.6, *p* = 0.95) or allograft survival (HR 1.73, 95% CI 0.23–13.23, *p* = 0.59) for those using steatotic grafts. Lipopeliosis on biopsy was common in those who developed AKI (61.0% vs. 31.6%, *p* = 0.04), particularly when the Model for End-Stage Liver Disease (MELD) was ≥20 (88.9%; *p* = 0.04). Lipopeliosis was a predictor of post-LT AKI (OR 6.0, 95% CI 1.1–34.6, *p* = 0.04). Conclusion: One-year outcomes for moderately steatotic grafts are satisfactory; however, a higher percentage of post-LT AKI and initiation of dialysis can be expected. Presence of lipopeliosis on biopsy appears to be predictive of post-LT AKI.

## 1. Introduction

Due to the ongoing organ shortage, there has been an increased interest in utilizing moderately steatotic donor liver allografts to maximize opportunities for transplantation. Historically, the use of liver allografts with significant steatosis has been associated with increased risk of primary nonfunction, poor early graft function, and decreased patient and allograft survival [1,2,3,4,5]. The degree of steatosis, as well as its histological pattern, appears to impact patient and allograft survival [2,6]. While allografts with severe macrovesicular steatosis (>60%) carry a very high risk of primary non-function, those with mild macrovesicular steatosis (<30%) yield results similar to those of non-steatotic liver allografts [2]. The outcomes of liver allografts with moderate steatosis (30% to 60%) remain variable and the impact of graft steatosis on renal function has not been well described [7,8]. As such, the objectives of this study were to: (1) evaluate postoperative acute kidney injury (AKI) patterns in recipients of steatotic grafts; (2) assess biopsy-findings predictive of AKI in the use of steatotic livers; (3) examine one-year patient and allograft outcomes.

## 2. Materials and Methods

### 2.1. Study Population

This was an eight-year, two-center retrospective study of patients who underwent a liver transplant at Mayo Clinic Arizona and Mayo Clinic Minnesota between January 2009 and December 2016 (*n* = 810). This study was approved by the Mayo Clinic Institutional Review Board. Outcomes were compared between moderately steatotic (>30% macrovesicular steatosis) and non-steatotic (<30% macrovesicular steatosis) grafts. In order to assess post-liver transplant (LT) acute kidney injury (AKI) patterns, patients with pre-LT acute kidney injury (AKI) (*n* = 143) were excluded. The majority of LT were performed via piggyback technique; 2.7% (*n* = 14) were performed via caval interposition. The data that supports the findings of this study is available from the corresponding author upon reasonable request. 

Pre-LT AKI was defined using the Kidney Disease Improving Global Outcomes (KDIGO) guidelines by an increase in serum creatinine > 0.3 mg/dL or 1.5–1.9 times baseline. Post-LT AKI was defined by those patients satisfying the KDIGO guidelines within 48 h post-LT and maintaining the criteria for > 48 h. Patients with pre-existing chronic kidney disease were not excluded unless they had pre-LT AKI. Mean follow-up was 4.2 years. 

All liver allografts were biopsied at the time of transplant and the biopsies were reviewed by a group of designated liver pathologists. Biopsy reports were retrospectively reviewed and macrovesicular steatosis percentage was recorded (less than 30% vs. greater than 30%). Early allograft dysfunction was defined as an aspartate aminotransferase (AST) greater than 2000 U/L within the first 7 days post-LT. 

Liver biopsies reported as having moderate (>30% macrovesicular) steatosis were prospectively re-reviewed by a single liver pathologist (M.S.) to specifically assess for macro- and microvesicular steatosis, the percentage of small- and large droplet macrovesicular steatosis, zonation, features of steatohepatitis, fibrosis, preservation reperfusion injury (PRI), and lipopeliosis. Percentages of steatosis were based on the percentage of hepatic parenchyma involved by fat and were estimated using previously published reference figures [9].

Operative hemodynamics were quantified using a modified operative inotrope score (inotrope score = dopamine (×1) + dobutamine (×1) + amrinone (×1) + milrinone (×15) + epinephrine (×100) + norepinephrine (×100) + phenylephrine (×1) + vasopressin (×1), with each drug dosed in μg/kg/min). The inotrope score was calculated during the pre-anhepatic, anhepatic, and post-reperfusion phases for the moderately macrovesicular steatosis cohort [10]. An average of systolic blood pressure (SBP) and mean arterial pressure (MAP) was also recorded for these operative periods. 

Immunosuppression was maintained per our center’s protocol. All recipients were started on mycophenolate mofetil (MMF), tacrolimus, and prednisone post-LT. MMF was withdrawn at 2–4 months; prednisone was discontinued at 4 months. Tacrolimus trough levels were set at 7–10 ng/mL for the first 2 months post-LT; at one-year post-LT, trough levels were reduced to 4–6 ng/mL. By protocol at our centers, tacrolimus is typically started immediately following transplant unless there are concerns for renal insufficiency. In the setting of post-LT renal insufficiency (defined as creatinine >2.0 mg/dL, estimated glomerular filtration rate (eGFR) <40 mL/minute or dialysis), Basiliximab 20 mg is given on post-operative days 0 and 4 to allow for a delay in tacrolimus initiation. In this setting, tacrolimus initiation is delayed until postoperative day 5. 

### 2.2. Statistical Methods

Descriptive analysis was performed using t-tests for continuous variables and Chi-square for categorical variables. Continuous variables are shown using mean and standard deviation; categorical variables using count and percentage. Wilcoxon rank-sum tests were used for non-normally distributed continuous variables. Survival analysis was performed using Kaplan-Meier analysis. Logistic regression was applied to clinically significant variables. Statistical analysis was performed using Prism software version 7.03 (La Jolla, CA, USA) and SAS version 9.4. A *p* value of <0.05 was considered significant.

## 3. Results

### 3.1. Postoperative Outcomes

**Figure 1:**
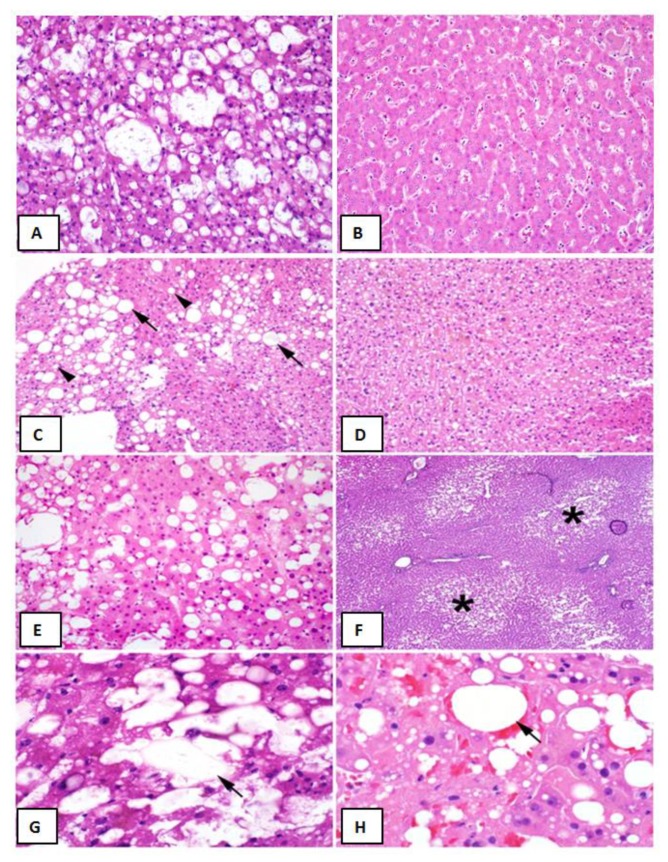
Liver Graft Biopsy Findings Liver Biopsy Findings. (**A**) Representative biopsy of a >30% macrovesicular steatotic allograft. (**B**) Representative biopsy of a non-steatotic allograft. (**C**) Approximately 30% macrovesicular steatosis with a mixture of large (arrows) and small (arrowheads) droplet fat (hematoxylin and eosin (H&E), 200×). (**D**) Microvesicular steatosis characterized by diffuse deposition of fat droplets in the hepatocyte cytoplasm without any macrovesicular steatosis (H&E, 200×). (**E**) Approximately 40% macrovesicular steatosis seen on a pre-implantation biopsy (H&E frozen section, 400×). (**F**) Zonal distribution of macrovesicular steatosis with fat deposition accentuated in zone three (asterisks) around the central veins (H&E, frozen section, 100×). (**G**), Lipopeliosis characterized by the rupture of hepatocytes with coalescence of fat droplets (arrow) in the sinusoidal spaces (H&E, frozen section, 600×). (**H**), Lipopeliosis (arrow) in post-reperfusion biopsy (H&E, 600×).

Of the 511 grafts that were included in this analysis, 40 were found to have moderate (>30%) macrovesicular steatosis. The average steatosis percentage was 41.1% ± 15.8% in the moderate group, compared to 3.8% ± 5.8% in the non-steatotic group (*p* < 0.001) (Table 1, Figure 1A,B). For the entire cohort, the incidence of post-LT AKI was 19.6% (*n* = 100). In assessing clinical risk factors for kidney disease, 12.5% (*n* = 64) of LT recipients were diabetic, 12.7% (*n* = 65) had hypertension, and 13.5% (*n* = 69) were both diabetic and hypertensive. The distribution of diabetes and hypertension did not vary between the steatotic and non-steatotic grafts (*p* = 0.95). There were no differences in age (*p* = 0.39), sex (*p* = 0.18), ethnicity (*p* = 0.72), race (*p* = 0.57), the biologic Model for End-Stage Liver Disease (MELD) (*p* = 0.14) or indications for liver transplant (*p* = 0.16) between the recipients receiving moderately steatotic and non-steatotic grafts (Table 1). 

Post-LT AKI was observed in 52.5% of patients receiving moderately steatotic grafts versus 16.8% in the non-steatotic cohort (*p* < 0.0001). No patients in the entire cohort had liver allograft primary non-function. Patients transplanted with moderately steatotic grafts had significantly more early allograft dysfunction immediately following surgery (AST: 3212 ± 2413 U/L vs. 1118 ± 1473 U/L, *p* < 0.0001). The rise in AST was four-fold higher for recipients of steatotic grafts that went on to develop post-LT AKI (*p* < 0.0001). There was a greater need for newly initiated temporary post-LT dialysis in the moderately steatotic group (10.0% vs. 1.1%, *p* = 0.003). There were no differences in intensive care unit (ICU) length of stay (2.0 ± 1.8 vs. 1.8 ± 2.6, *p* = 0.62) or total hospital length of stay (9.1 ± 10.3 vs. 9.9 ± 10.9, *p* = 0.67). At one-year post-LT, there were no observed differences in the need for new chronic (ongoing) post-LT dialysis (*p* > 0.99), serum creatinine (1.3 ± 0.3 vs. 1.3 ± 0.7, *p* = 0.97), or eGFR (53.1 ± 7.9 vs. 53.5 ± 10.1, *p* = 0.70) (Table 1).

### 3.2. Moderately Steatotic Graft Subgroup Analysis

In order to investigate postoperative outcomes in moderately steatotic livers allografts in further detail, we reviewed the characteristics of the 40 patients who were transplanted with such grafts (Table 2). In this cohort, there were no differences in age (*p* = 0.61), sex (*p* = 0.43), ethnicity (*p* = 0.60), race (*p* = 0.64), or indication for transplant (*p* = 0.53) among those who developed AKI versus those who did not (Table 2). Of those receiving steatotic grafts, 52.5% went on to develop AKI; the other 47.5% maintained normal renal function post-transplant. Recipients of steatotic grafts that went on to develop post-LT AKI had a higher biologic MELD (20.5 ± 8.9 vs. 15.3 ± 6.9, *p* = 0.04) (Table 2). There were no differences in pre-LT creatinine (*p* = 0.21) or eGFR (*p* = 0.88). 

**Figure 2:**
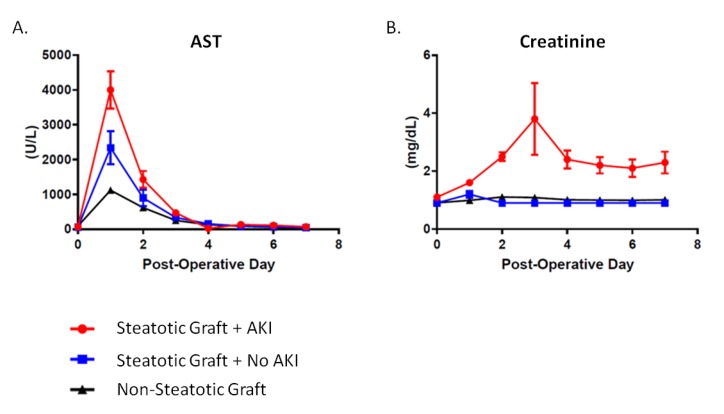
Post-Liver Transplant Patterns in Steatotic and Non-Steatotic Grafts. (**A**) Post-LT aspartate aminotransferase (AST) levels. Compared to non-steatotic graft, AST levels were 2-times higher in steatotic grafts without post-LT AKI and 4-times higher for steatotic grafts with AKI. (**B**) Post-LT creatinine levels.

Recipients of moderately steatotic grafts were all noted to have early allograft dysfunction as demonstrated through a significantly elevated AST immediately following surgery (Figure 2). This occurred regardless of whether or not they developed AKI. The rise in AST was four-fold higher for recipients of steatotic grafts that went on to develop post-LT AKI as compared to non-steatotic grafts (40001.0 ± 2471.0 U/L vs. 1118.0 ± 1473.0, *p* < 0.0001). The rise in AST was also two-fold higher when comparing steatotic grafts of recipients with and without post-LT AKI (40001.0 ± 2471.0 U/L vs. 2339.0 ± 2074.0, *p* < 0.0001). There were no differences between the post-LT AKI and no AKI groups with regards to graft type (i.e., donation after brain death, DBD, vs. donation after cardiac death, DCD) (19.0% vs. 10.5%, *p* = 0.66), sex (female: 42.9% vs. 47.4%, *p* = 0.38), or BMI (32.8 ± 5.9 kg/m^2^ vs. 32.6 ± 9.4 kg/m^2^, *p* = 0.92). Donor age was yonger (43.2 ± 12.6 vs. 52.8 ± 14.9%, *p* = 0.03) in steatotic grafts that went on to develop AKI. In addition, there were no differences in allograft cold ischemia time (CIT) (*p* = 0.28) or estimated operative blood loss (EBL) (*p* = 0.49) (Table 3). 

When operative hemodynamics of AKI and non-AKI recipients receiving moderately steatotic grafts were compared, there were no differences observed in inotrope requirements (*p* = 0.45), systolic blood pressure (SBP) (*p* = 0.25) or mean arterial pressure (MAP) (*p* = 0.76) during the pre-anhepatic phase or anhepatic phase (inotrope score *p* = 0.58; SBP *p* = 0.67; MAP *p* = 0.71) of the operation (Table 3). Post-reperfusion, SBP (*p* = 0.87) and MAP (*p* = 0.69) were similar in patients who went on to develop post-LT AKI versus those who did not suggesting appropriate perfusion parameters were able to be maintained. The post-reperfusion inotrope requirements, however, were significantly higher in the post-LT AKI group (19.5 ± 20.0 vs. 3.8 ± 4.4, *p* = 0.03). Ten percent of the patients in the steatotic post-LT AKI group (*n* = 4) required initiation of new dialysis post-LT (*p* = 0.003) (Table 2). There were no differences in ICU length of stay (*p* = 0.62) and total hospital length of stay (*p* = 0.67) between steatotic AKI and no AKI groups.

**Figure 3:**
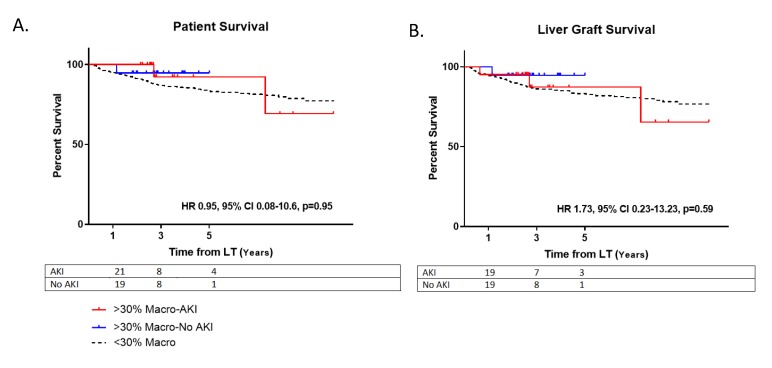
Survival. (**A**) Patient survival post-LT. Hazard ratio (HR); Confidence Interval (CI). (**B**) Liver allograft survival post-transplant. Moderately steatotic grafts with AKI (>30% Macro-AKI); Moderately steatotic grafts without AKI (>30% Macro-No AKI); Non-steatotic grafts (<30% Macro).

At one-year post-LT, there were no observed differences between those receiving steatotic grafts that developed AKI versus those who did not with regards to serum creatinine (1.3 ± 0.2 vs. 1.2 ± 0.4, *p* = 0.27) and eGFR (52.7 ± 6.9 vs. 53.6 ± 9.1, *p* = 0.74) (Table 2). At five years, there were no differences for AKI vs. no AKI patient survival (HR 0.95, 95% CI 0.08–10.6, *p* = 0.95) or allograft survival (HR 1.73, 95% CI 0.23–13.23, *p* = 0.59) for those using steatotic grafts (Figure 3).

### 3.3. Liver Graft Biopsy Findings

**Figure 4:**
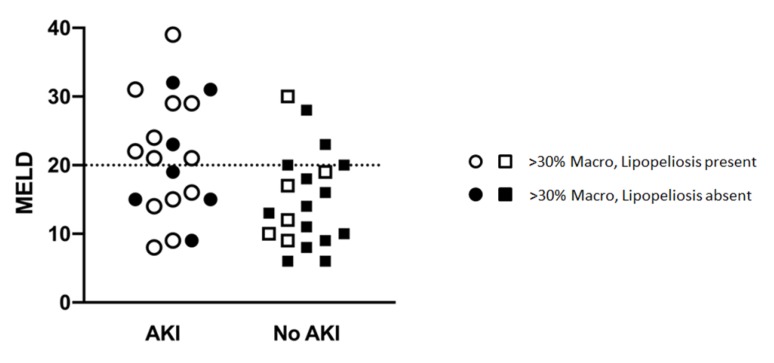
Relationship between MELD, Lipopeliosis and Post-LT AKI.

In prospectively re-reviewing biopsies of all liver allografts with moderate (>30%) macrovesicular steatosis, the majority of the steatosis was found to be large droplet (Table 4) (Figure 1C,E). When comparing biopsies in patients with and without post-LT AKI, no differences were observed with regard to large droplet versus small droplet percentage composition (Figure 1C) (*p* = 0.41). Although microvesicular steatosis was minimal in both groups (Figure 1D) (0.0% vs. 21.1%), a higher frequency was observed in the no AKI group (*n* = 4, *p* = 0.04) (Table 4). No significant differences were observed in the histologic distribution of the steatosis (zonation) in the allograft (*p* = 0.75) (Figure 1F), inflammation (*p* = 0.73), ballooning (*p* = 0.65), or Mallory hyaline (Table 4). A larger percentage of biopsies in the post-LT AKI group contained lipopeliosis (61% vs. 31.6%, *p* = 0.04) (Figure 1G–H). When plotted against MELD at the time of transplant, recipients of moderately steatotic grafts with lipopeliosis with a MELD ≥ 20 were found to more likely to develop AKI (88.9%) than recipients of such grafts with MELD < 20 (40.0%; *p* = 0.04) (Figure 4). In using logistic regression, variables predictive of post-LT AKI included the finding of lipopeliosis on liver biopsy and donor age (Table 5).

## 4. Discussion

Primary nonfunction, poor early graft function, and decreased patient and allograft survival have all been associated with the use of steatotic liver allografts for transplantation [1,2,3,5]. This, combined with inaccurate and inconsistent reporting of liver allograft biopsies, has led to a high discard rate of grafts with moderate (>30%) steatosis [11]. While the risk of adverse events with severely steatotic liver allografts (>60%) remains well recognized, the use of moderately steatotic grafts (30%–60%) has been increasing [7]. 

It has been our experience that steatotic grafts almost universally exhibit early allograft dysfunction and require additional resource utilization postoperatively specific to the development of post-LT AKI and the need to initiate new dialysis [7,12,13,14,15]. In this study, the occurrence of AKI post-LT was noted to be markedly increased at 52.5% compared to 19.6% observed in the general liver transplant recipient pool. Although early allograft dysfunction, best demonstrated by significantly elevated transaminases, was common to all steatotic grafts, not all recipients developed post-LT AKI (Figure 2). We have clinically observed this divergent pattern; however, it remains difficult to quantify why some steatotic grafts behave in this manner while others do not. In this study, patients receiving steatotic grafts that developed post-LT AKI were noted to have higher inotrope requirements post-reperfusion. Despite having higher inotrope requirements, no differences were observed in hemodynamic parameters (systolic blood pressure and MAP) between the patients with and without post-LT AKI, suggesting that variables other than hemodynamics influence the development of AKI. Not surprisingly, a higher MELD score was associated with an increased risk of post-LT AKI. This association was particularly strong when steatotic grafts with lipopeliosis on biopsy were transplanted to patients with MELD scores of 20 or above (Table 2 and Table 5) (Figure 4). 

Proper classification of graft steatosis remains challenging even within the transplant community [9]. Historically, steatosis was classified as microvesicular or macrovesicular, based on hepatocyte fat droplet size and nucleus displacement. Although often reported, true microvesicular steatosis is rare and manifests histologically as diffuse deposition of small lipid droplets in the hepatocyte cytoplasm with a resulting foamy appearance (Figure 1D). Two types of fat droplets are seen in the setting of macrovesicular steatosis: small droplets and large droplets (Figure 1C). Fat droplets in small droplet steatosis are not large enough to displace the nucleus; this finding is often inaccurately reported as being microvesicular steatosis on biopsy. Although both small and large droplet steatosis contribute to overall macrovesicular steatosis, historically small droplet macrovesicular steatosis and microvesicular steatosis were used synonymously, resulting in ongoing confusion [3]. We hypothesize that some of the observed differences in post-LT AKI between otherwise similarly-appearing steatotic grafts might be related to this histological variation. 

The term lipopeliosis was first identified in the early days of hepatic transplantation and describes the coalescence of fat droplets from ruptured hepatocytes into larger droplets of fat in the sinusoidal space [16]. Due to the universal finding of preservation-related injury in these biopsies, lipopeliosis was presumed to not be of clinical significance [17]. In our experience, however, the histologic finding of lipopeliosis is, by far, more common in patients who develop post-LT AKI and, in our experience, has been associated with inferior post-transplant outcomes [18]. 

Although the current study was not designed to elucidate the mechanisms of AKI post-LT, we have previously shown that fat droplets, through the process of lipopeliosis, embolize to the pulmonary vasculature following reperfusion with resulting respiratory failure [18]. This mechanism is likely similar to that seen after long bone traumatic injuries, where fat droplets are released into the venous system and migrate to the pulmonary capillary beds [19,20]. Microvascular lodging results in ischemia, inflammation, and release of inflammatory mediators. The breakdown of fat emboli by pneumocytes can results in release of free fatty acids that, in turn, enter systemic circulation and result in multisystem dysfunction [21]. The finding of fat droplets in the urine under these circumstances correlates with the development of AKI and a systemic process triggered by fat embolization [22]. Whether lipopeliosis in steatotic liver graft biopsies can be used to predict clinical instability and the development of post-LT AKI will need to be validated in a larger prospective study. Limitations to this study include its overall small cohort size. The results also represent the experience of only two centers. Patients with pre-LT AKI were also excluded in this study to better assess post-LT outcomes specific to AKI development. The impact of steatosis on renal function in patients with preexisting AKI remains uncertain. 

In conclusion, utilization of moderately steatotic grafts is associated with a significantly higher risk for developing post-LT AKI. This risk appears to be independent of pre-reperfusion operative hemodynamics. In utilizing these grafts, laboratory abnormalities persist 2 to 3 months post-LT, but there does not appear to be an impact on long-term renal function, patient, or graft survival. The risk for AKI in this setting appears increased when the MELD score is greater than 20 and lipopeliosis is histologically present on biopsy. These outcomes are more favorable as compared to older studies, and suggest that, with lower MELD recipients, satisfactory outcomes can be achieved with the use of these grafts [5]. 

## Figures and Tables

**Table 1:** Pre- and Post-Liver Transplant Recipient Demographics.

	Steatotic Grafts (*n* = 40)	Non-Steatotic Grafts (*n* = 471)	*p* Value
**Pre-Liver Transplant**			
Steatosis (%)	41.1 ± 15.8	3.8 ± 5.8	<0.0001
Recipient Age (years)	57. 4 ± 9.7	56.0 ± 10.3	0.39
Biologic MELD	18.0 ± 8.3	16.1 ± 7.0	0.14
Female	9 (22.5%)	155 (32.9%)	0.18
**Indication for LT**			0.16
Hepatitis C (HCV)	6 (22.5%)	55 (11.6%)	
Cholestatic	5 (12.5%)	53 (11.3%)	
Hepatocellular Carcinoma (HCC)/Other MELD Exception	11 (27.5%)	223 (47.3%)	
Alcohol-Related Liver Disease (ALD)	8 (20.0%)	46 (9.8%)	
Nonalcoholic Steatohepatitis (NASH)	6 (15.0%)	49 (10.4%)	
Other	4 (10.0%)	45 (9.6%)	
Pre-LT Creatinine (mg/dL)	1.0 ± 0.3	0.9 ± 0.9	0.14
Pre-LT eGFR	57.7 ± 6.3	57.7 ± 6.6	0.97
**Post-Liver Transplant**			
Post-LT AKI	21 (52.5%)	79 (16.8%)	<0.0001
Post-LT Temporary Dialysis	4 (10.0%)	5 (1.1%)	0.003
ICU LOS (days)	2.0 ± 1.8	1.8 ± 2.6	0.62
Total Hospital LOS (days)	9.1 ± 10.3	9.9 ± 10.9	0.67
**One-Year Post-Liver Transplant**			
Creatinine (mg/dL)	1.3 ± 0.3	1.3 ± 0.7	0.97
eGFR (mL/min)	53.1 ± 7.9	53.5 ± 10.1	0.70
New Chronic Post-LT Dialysis	0 (0.0%)	2 (0.4%)	>0.99

**Table 2:** Moderately Steatotic Graft Subgroup Analysis.

	Post-LT AKI (*n* = 21)	No AKI (*n* = 19)	*p* Value
**Pre-Liver Transplant**			
Macrovesicular Steatosis (%)	41.9 ± 15.7	40.3 ± 16.2	0.75
Recipient Age (years)	56.7 ± 8.3	58.3 ± 11.3	0.61
Biologic MELD	20.5 ± 8.9	15.3 ± 6.9	0.04
Female	3 (14.3%)	6 (31.6%)	0.43
**Indication for LT**			0.53
HCV	4 (19.0%)	2 (10.5%)	
Cholestatic	1 (4.8%)	4 (21.1%)	
HCC/Other MELD Exception	5 (23.8%)	6 (31.6%)	
ALD	4 (19.0%)	4 (21.1%)	
NASH	4 (19.0%)	2 (10.5%)	
Other	3 (14.3%)	1 (5.3%)	
Total Hospital LOS (median)	10.4 ± 13.5 (7.0)	7.1 ± 4.1 (6.0)	0.32
**One-Year Post-LT**			
Creatinine (mg/dL)	1.3 ± 0.2	1.2 ± 0.4	0.27
eGFR (mL/min)	52.7 ± 6.9	53.6 ± 9.1	0.74

**Table 3:** Steatotic Graft Recipient Operative Variables.

	Post-LT AKI (*n* = 21)	No AKI (*n* = 19)	*p* Value
CIT (h)	6.8 ± 2.1	6.1 ± 1.7	0.28
EBL (mL)	2157 ± 1649	1821 ± 1405	0.49
Pre-Anhepatic			
SBP (mmHg)	111.2 ± 21.8	104.2 ± 14.5	0.25
MAP	75.1 ± 13.3	73.8 ± 13.6	0.76
Inotrope Score	4.1 ± 11.9	1.7 ± 6.9	0.45
Anhepatic			
SBP (mmHg)	106.5 ± 15.4	104.4 ± 15.1	0.67
MAP	75.4 ± 11.0	76.7 ± 9.6	0.71
Inotrope Score	4.3 ± 11.9	2.6 ± 6.8	0.58
Post Reperfusion			
SBP (mmHg)	95.6 ± 13.8	96.2 ± 7.9	0.87
MAP	67.0 ± 11.8	65.5 ± 9.8	0.69
Inotrope Score	19.5 ± 20.0	3.8 ± 4.4	0.03

**Table 4:** Steatotic Liver Graft Biopsy Findings.

	Post-LT AKI (*n* = 21)	No AKI (*n* = 19)	*p* Value
Macrovesicular Steatosis			
Large Droplet (%)	69.2 ± 16.1	64.7 ± 16.9	0.41
Small Droplet (%)	30.8 ± 16.1	35.3 ± 16.9	
Microvesicular Steatosis	0 (0.0%)	4 (21.1%)	0.04
Zonation	14 (66.7%)	11 (57.9%)	0.75
Inflammation	5 (23.8%)	6 (31.5%)	0.73
Ballooning	2 (9.5%)	3 (15.8%)	0.65
Mallory Hyaline	0 (0.0%)	0 (0.0%)	-
Lipopeliosis	13 (61.9%)	6 (31.6%)	0.03

**Table 5:** Predictors of AKI in Steatotic Grafts—Logistic Regression.

Effect	Odds Ratio	95% CI		*p* Value
Donor Age	0.93	0.87	0.99	0.02
Lipopeliosis	6.04	1.05	34.61	0.04
Post-Reperfusion Systolic BP	1.01	0.95	1.08	0.75
